# Pineal gland cyst and ADHD in a juvenile patient: a case report

**DOI:** 10.1192/j.eurpsy.2024.969

**Published:** 2024-08-27

**Authors:** A. Kovačević, N. Rančić, T. Mastelić, M. Ćurković, I. Barišić, I. Lozina, M. Rogulj, D. Lasić, T. Glavina

**Affiliations:** Psychiatry Department, University Hospital of Split, Split, Croatia

## Abstract

**Introduction:**

The role of the pineal gland in psychiatric disorders is poorly investigated. There are studies, primarily on adult patients, that indicate a higher presence of pineal gland abnormalities in patients suffering from depression, schizophrenia and attention-deficit/hyperactivity disorder (ADHD). When it comes to ADHD, there is speculation about the role of melatonin and the influence of the pineal gland on the dopaminergic system. Data on the association between pineal gland cysts and ADHD in juvenile patients are particularly scarce.

**Objectives:**

Due to all of the above, our goal is to present the case of a nine-year-old male patient who has a confirmed cyst of the pineal gland and is being treated for ADHD.

**Methods:**

The patient was examined by a neuropediatrician, EEG and brain MRI were performed. He was also examined by a psychologist and by a psychiatrist. Endocrinological, hematological, rheumatological, pulmonological treatment and karyotyping were performed.

**Results:**

MRI of the brain revealed a cyst of the pineal gland with an anteroposterior diameter of 1 cm without significant compression. The EEG was mildly slowed and paroxysmally dysrhythmic for the age, ie. paroxysms of high-voltage delta waves were described. The EEG findings after sleep deprivation were paroxysmally altered with rare focal changes in the right temporoparietal region. Through psychological analysis, it was determined that specific deficits persist in the area of verbal understanding, perceptual organization and visual processing, information processing speed, numerical reasoning, attention and short-term memory. On the level of visuomotor perception and coordination, deviations are observed by organic type. He is motorically more active, impulsive, emotionally immature, easily distractible.

**Conclusions:**

The etiology of ADHD is poorly researched, and so is the role of the pineal gland, its cyst and melatonin. There is scant knowledge for other psychiatric disorders, but primarily from researches on adult psychiatric patients. Additional researches are definitely needed on this topic, especially in the field of child and adolescent psychiatry.

**Disclosure of Interest:**

None Declared

